# Stiffness-Modulation of Collagen Gels by Genipin-Crosslinking for Cell Culture

**DOI:** 10.3390/gels9020148

**Published:** 2023-02-10

**Authors:** Seiichiro Ishihara, Haruna Kurosawa, Hisashi Haga

**Affiliations:** 1 Department of Advanced Transdisciplinary Sciences, Faculty of Advanced Life Science, Hokkaido University, N10-W8, Kita-ku, Sapporo 060-0810, Japan; 2 Division of Soft Matter, Graduate School of Life Science, Hokkaido University, N10-W8, Kita-ku, Sapporo 060-0810, Japan

**Keywords:** collagen gel, extracellular matrix, stiffness, genipin, cell culture, cell morphology, YAP, mesenchymal stromal cells, cancer cells, differentiation

## Abstract

The stiffness of extracellular matrices (ECMs) is critical for cellular functions. Therefore, modulating the stiffness of ECMs in vitro is necessary to investigate the role of stiffness in cellular phenomena. Collagen gels are widely used for cell culture matrices in vitro. However, modulation of the stiffness in collagen gels for cell culture is challenging owing to the limited knowledge of the method to increase the stiffness while maintaining low cytotoxicity. Here, we established a novel method to modulate collagen gel stiffness from 0.0292 to 12.5 kPa with low cytotoxicity. We prepared collagens with genipin, a low-cytotoxic crosslinker of amines, at different concentrations and successfully modulated the stiffness of the gels. In addition, on 10 mM genipin-mixed collagen gels (approximately 12.5 kPa), H1299 human lung cancer cells showed spreading morphology and nuclear localization of yes-associated protein (YAP), typical phenomena of cells on stiff ECMs. Mouse mesenchymal stromal cells on 10 mM genipin-mixed collagen gels differentiated to vascular smooth muscle cells. On the other hand, the cells on 0 mM genipin-mixed collagen gels (approximately 0.0292 kPa) differentiated to visceral smooth muscle cells. Our new method provides a novel way to prepare stiffness-modulated collagen gels with low cytotoxicity in cell culture.

## 1. Introduction

Cells respond to mechanical stress and regulate their phenotype. In particular, once cells adhere to extracellular matrices (ECMs), they sense their stiffness and determine their fate [1]. For instance, fibroblasts easily migrate from soft ECMs to stiff ECMs, while it is difficult for cells to move from stiff ECMs to soft ECMs [2]. The morphology of fibroblasts is also regulated by ECM stiffness, as stiff ECMs induces spreading morphology [3]. In addition, epithelial cell clusters on stiff ECMs show random migration, whereas those on soft ECMs display collective migration [4]. Mesenchymal stromal/stem cells (MSCs) are multipotent mesenchymal cells that exist in many types of tissues [5] and regulate their differentiation potential by stiffness of the surrounding matrix. A previous study reported that soft ECMs, ECMs with moderate stiffness, and stiff ECMs trigger the neurogenic, myogenic, and osteogenic differentiation of MSCs, respectively [6]. Neural stem cells also respond to matrix stiffness and differentiate into neurons and astrocytes [7]. Furthermore, ECM stiffness is critical for cancer progression. Most solid cancers have stiff tissues, which affect the biochemical signaling and phenotypes of the cells in the tumors [8,9,10]. Stiff ECMs activate transcription factors, such as nuclear factor-κB (NF-κB), twist family bHLH transcription factor 1 (Twist1), and yes-associated protein (YAP) [11,12,13], thereby promoting malignant phenotypes in cancer cells. Stroma cells, such as cancer-associated fibroblasts (CAFs) in tumors, also respond to ECM stiffness and modulate cancer progression [14,15]. Therefore, the stiffness of the ECMs contributes to cellular functions and phenomena.

To evaluate the role of ECM stiffness in cell fate in vitro, we cultured the cells on ECMs with different stiffnesses. Collagens, one of the major components of ECMs [16], are widely used for cell culture. For example, the combination of a stiff substrate (collagen-coated glass or plastic dish) and a soft substrate (collagen gel) is one of the conventional methods used to determine the effect of ECM stiffness on cells [4,11,17]. In addition, by changing the collagen concentration, we can obtain gels with different stiffnesses, as higher collagen concentrations result in stiffer gel properties [8,18]. These methods are convenient; however, the exact concentration of collagen in these substrates cannot be controlled. Therefore, we cannot eliminate the biochemical effects of collagen molecules owing to differences in collagen concentration. Another way to prepare substrates with different stiffnesses is to use polyacrylamide gels with different crosslinker concentrations [19,20]. In this way, we can coat the gels with appropriate concentrations of ECMs; however, some cells can degrade the coated ECMs and, as a result, the artificial polyacrylamide gel surface is directly exposed to the cells. Another alternative method is crosslinking collagen gels with crosslinkers, such as N-(3-Dimethylaminopropyl)-N′-ethylcarbodiimide hydrochloride (EDC); however, using these crosslinkers poses a risk of cytotoxicity [21]. Although chitosan–hyaluronic acid dialdehyde hydrogels with different stiffnesses are used as substrates for cell culture [22], they are not derived from mammalian ECMs. Thus, a new way to control the stiffness of ECMs, such as collagen gels with the same concentration of ECMs and low cytotoxicity, is urgently needed.

Genipin is reported to be a crosslinker of amines in collagens [23,24] and a low cytotoxic reagent to cells [25]. Therefore, we used genipin to prepare collagen gels with different stiffness values for the cell culture. We successfully modulated the stiffness of collagen gels from 0.0292 to 12.5 kPa with low cytotoxicity. Moreover, we confirmed that the cells responded to the stiffness of these gels, as the H1299 lung cancer cells showed spreading morphology and nuclear localization of YAP, typical phenomena of cells on a stiff substrate. Furthermore, MSCs on stiff genipin-mixed collagen gels differentiated into vascular smooth muscle cells, whereas MSCs on soft collagen gels differentiated into visceral smooth muscle cells. These results suggest that our novel method for preparing stiffness-modulated collagen gels is a powerful tool for evaluating the effects of ECM stiffness on cultured cells in vitro.

## 2. Results and Discussion

### 2.1. Preparing Collagen Gels with Different Stiffness by Genipin-Crosslinking

**Figure 1:**
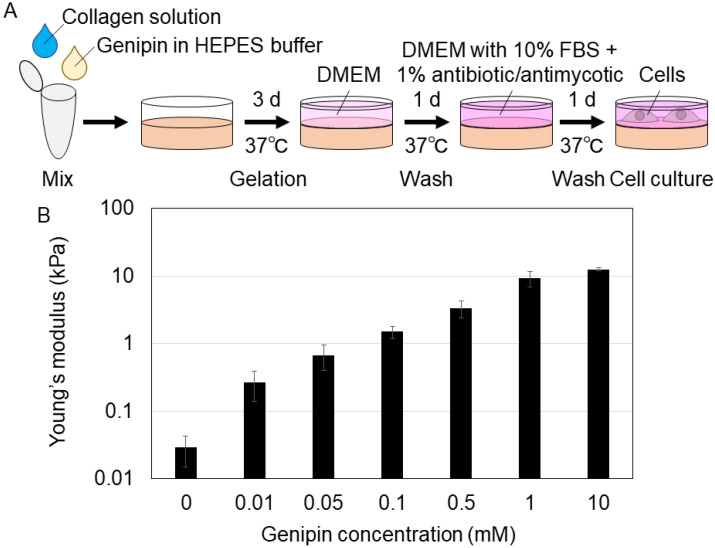
(**A**) The schematic procedure of preparing genipin-mixed collagen gels for cell culture. HEPES: N-2-hydroxyethylpiperazine-N-2-ethane sulfonic acid, DMEM: Dulbecco’s modified Eagle’s medium, FBS: fetal bovine serum. (**B**) Young’s moduli of the 0, 0.01, 0.05, 0.1, 0.5, 1, or 10 mM genipin-mixed collagen gels. Mean ± S.D. *N* = 3 experiments.

First, we prepared collagen gels with genipin crosslinking to prepare gels with different stiffness values. We mixed a collagen solution and genipin-mixed HEPES (N-2-hydroxyethylpiperazine-N-2-ethane sulfonic acid) buffer and incubated the solution for gelation (Figure 1A). Collagen gels were mixed with 0, 0.01, 0.05, 0.1, 0.5, 1, or 10 mM genipin (final concentration). After washing the gels, we evaluated their stiffness (Young’s modulus) using atomic force microscopy. The Young’s moduli of the gels with 0, 0.01, 0.05, 0.1, 0.5, 1, and 10 mM genipin were 0.0292, 0.267, 0.678, 1.49, 3.36, 9.20, 12.5 kPa (mean value), respectively (Figure 1B). In addition, the genipin concentration in the media incubated for 24 h with 10 mM genipin-mixed collagen gel after washing was approximately 0.035 mM (determined by 595 nm absorbance). The cell viability of H1299 human lung cancer cell line and mouse mesenchymal stromal/stem cells (MSCs) was almost 100% with genipin concentrations less than 0.6 and 0.3 mM, respectively (Figure S1). Therefore, we confirmed that even at the highest genipin concentration in our experiments, the cytotoxicity of genipin derived from the genipin-mixed collagen gels was small enough to be ignored. As mentioned above, we successfully established a new method to prepare genipin-mixed collagen gels with different stiffnesses (0.0292–12.5 kPa) for cell culture.

### 2.2. Morphology of the Cells on Genipin-Mixed Collagen Gels

**Figure 2:**
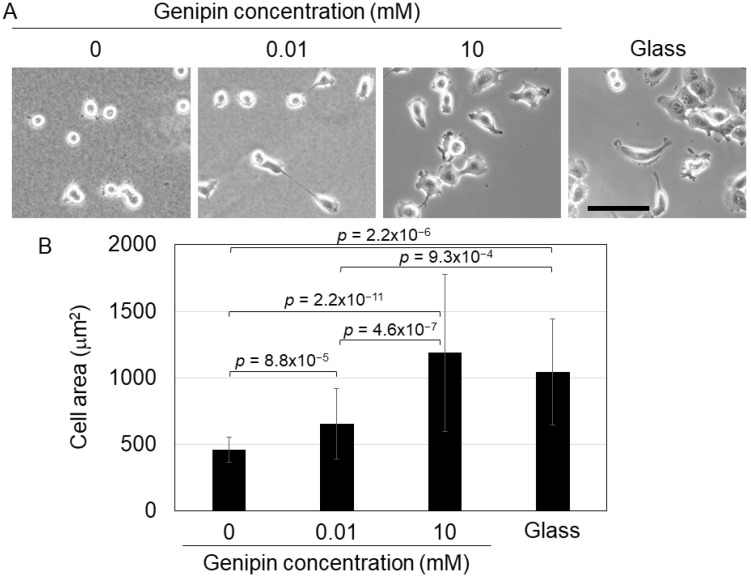
(**A**) Cell morphology of H1299 cells on the 0, 0.01, or 10 mM genipin-mixed collagen gels or collagen-coated glass substrates. Scale bar = 100 μm. (**B**) Cell area of H1299 cells shown in (**A**). Mean ± S.D. *N* = at least 23 cells in 3 independent experiments. *p* value was calculated using Welch’s *t*-test with Bonferroni correction.

Next, we cultured H1299 cells on genipin-mixed collagen gels and investigated their phenotype. A previous study showed that cultured cells, including H1299 cells, display spreading morphology on a stiff substrate, whereas on a soft substrate, the cells maintained a round morphology [3,11]. Therefore, we cultured H1299 cells on genipin-mixed collagen gels with different stiffnesses and a collagen-coated glass dish (for the control of a stiff substrate). Our results confirmed that H1299 cells on 0 mM genipin-mixed collagen gels (0.0292 kPa gels) displayed a round shape; in contrast, the cells on 10 mM genipin-mixed collagen gels (12.5 kPa) and glass dishes showed spreading morphology (Figure 2A). The cells on 0.01 mM genipin-mixed collagen gels (0.267 kPa gels) induced moderate spreading (Figure 2A). Cell area analysis confirmed that cell spreading was significantly induced on stiff substrates (Figure 2B). These results showed that H1299 cells responded to the stiffness of genipin-mixed collagen gels and changed their morphology.

### 2.3. YAP Localization of the Cells on Genipin-Mixed Collagen Gels

**Figure 3:**
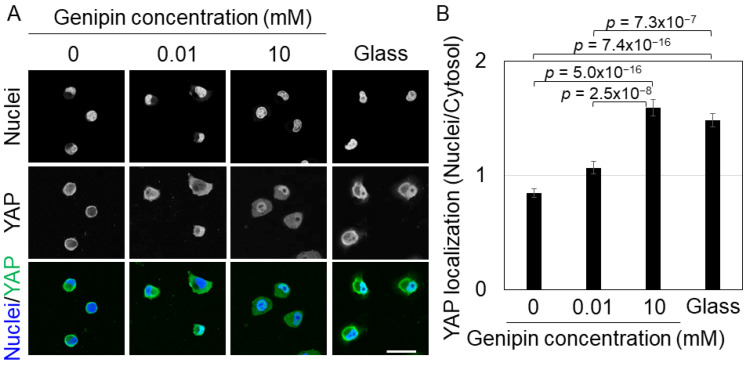
(**A**) Fluorescent staining of nuclei and YAP of H1299 cells on the 0, 0.01, or 10 mM genipin-mixed collagen gels or collagen-coated glass substrates. Scale bar = 50 μm. (**B**) YAP localization of H1299 cells shown in (**A**). Mean ± S.E. *N* = at least 84 cells in 4 independent experiments. *p* value was calculated using Welch’s *t*-test with Bonferroni correction.

YAP is a transcription factor that is localized to the nuclei of cells on a stiff substrate, whereas it diffuses or localizes to the cytoplasm of cells on a soft substrate [26]. Therefore, we cultured H1299 cells on genipin-mixed collagen gels and collagen-coated glass dishes and observed YAP localization using immunofluorescence staining. On 0 or 0.01 mM genipin-mixed collagen gels, YAP diffused in most cells (Figure 3A). In contrast, YAP localization to the nuclei was strongly induced in cells on 10 mM genipin-mixed collagen gels or glass substrates (Figure 3A). Statistical analysis of YAP nuclear localization showed that YAP was significantly localized to the nuclei of cells on 10 mM genipin-mixed collagen gels and glass dishes (Figure 3B). These results indicate that, similar to cell morphology, H1299 cells respond to the stiffness of genipin-mixed collagen gels and regulate YAP localization.

### 2.4. Differentiation of the MSCs on Genipin-Mixed Collagen Gels

**Figure 4:**
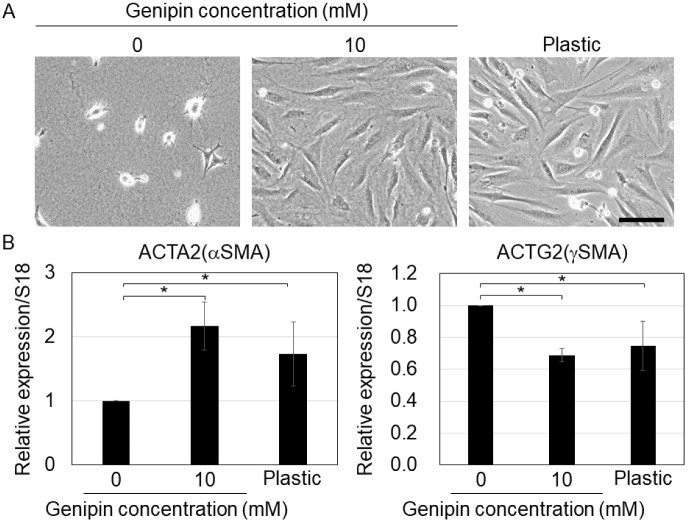
(**A**) Cell morphology of mouse mesenchymal stromal cells on the 0 or 10 mM genipin-mixed collagen gels or collagen-coated plastic substrates with smooth muscle-differentiation condition. Scale bar = 100 μm. (**B**) qPCR of ACTA2 and ACTG2 in (**A**). S18 was used as an internal control. Mean ± S.D. *N* = 3 independent experiments. * Statistical significance determined with 95% confidence interval with Bonferroni correction.

Previous studies have shown that ECM stiffness is critical for the differentiation potential of MSCs [6,26]. Thus, we cultured MSCs on genipin-mixed collagen gels with different stiffnesses and evaluated their differentiation potential. First, we cultured MSCs on genipin-mixed collagen gels and collagen-coated plastic dishes (to control stiff substrates) under smooth muscle differentiation conditions. Our results showed that on 10 mM genipin-mixed collagen gels or plastic substrates, the cells showed spreading morphology, whereas on 0 mM genipin-mixed collagen gels, the cells did not (Figure 4A), suggesting that MSCs responded to the stiffness of each substrate. The qPCR results revealed that ACTA2 (αSMA, a marker of vascular smooth muscle [27]) expression was significantly higher in the cells on 10 mM genipin-mixed collagen gels or plastic dishes, on the other hand, ACTG2 (γSMA, a marker of visceral smooth muscle [27]) expression was significantly lower in the cells on 10 mM genipin-mixed collagen gels or plastic dishes (Figure 4B). We also confirmed that the smooth muscle marker smoothelin (SMTN) [27] was expressed in cells on all substrates, even though the expression level was significantly higher in cells on 0 mM genipin-mixed collagen gels (Figure S2).

A previous study reported that in smooth muscle cells, high ACTA2 and low ACTG2 expressions are markers of vascular smooth muscle, whereas low ACTA2 and high ACTG2 expressions are markers of visceral smooth muscle [27]. Therefore, our results suggest that stiff substrates promote vascular smooth muscle differentiation and repress the visceral smooth muscle differentiation of MSCs. Although the mechanism that regulates the differentiation of MSCs into vascular or visceral smooth muscle is poorly understood, a previous study have shown that ACTA2 expression is upregulated by stiff substrates via YAP activation [15]. In addition, ACTA2-positive smooth muscle differentiation was reported to be promoted by the blockage of ERK signaling [28] and inhibited by miR-222-5p [29]. Therefore, stiff substrates may induce the differentiation of MSCs into vascular smooth muscles via the regulation of YAP, ERK, and miRNAs.

**Figure 5:**
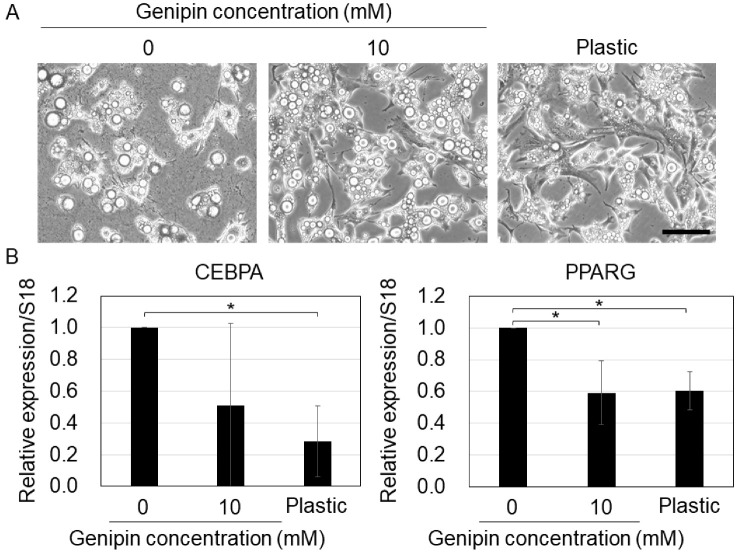
(**A**) Cell morphology of mouse mesenchymal stromal cells on the 0 or 10 mM genipin-mixed collagen gels or collagen-coated plastic substrates with adipogenic condition. Scale bar = 100 μm. (**B**) qPCR of CEBPA and PPARG in (**A**). S18 was used as an internal control. Mean ± S.D. *N* = 3 independent experiments. * Statistical significance determined with 95% confidence interval with Bonferroni correction.

Next, we cultured MSCs on genipin-mixed collagen gels and collagen-coated plastic dishes under adipogenic conditions. On softer substrates, the cells stored larger lipid droplets (Figure 5A). A previous study found that lipid droplets grow large during the differentiation process [30], implying that soft substrates enhance the differentiation of MSCs into adipocytes. The qPCR results showed that the adipogenic markers (CEBPA and PPARG) [31] were downregulated in cells on stiff substrates (10 mM genipin-mixed collagen gels and plastic dishes). A previous study also showed that adipogenic differentiation is promoted in MSCs cultured on soft substrates [26]. Therefore, our results indicate that genipin-mixed collagen gels with different stiffnesses also regulate adipogenic differentiation of MSCs.

### 2.5. Growth and Survival of the Cells on Genipin-Mixed Collagen Gels

We have previously shown that stiff ECMs promote growth of colon cancer cells [12] and MSCs in cancer-conditioned media [15]. In addition, hydrogel crosslinking affects gel porosity and cell proliferation [32]. Therefore, we examined the effect of hydrogel stiffness on the growth of cultured H1299 cells and MSCs by culturing them on genipin-mixed collagen gels of different stiffness. The results showed no significant differences in cell growth between the cells on 0 mM genipin-mixed collagen gels, 10 mM genipin-mixed collagen gels, and collagen-coated plastic substrates (Figure S3A,B). Cell survival was approximately 100% on all the substrates (Figure S3A,B), implying that cell growth reflects their proliferation. These results suggest that substrate stiffness and gel porosity are not critical for the proliferation of H1299 cells or MSCs.

These results demonstrate the successful preparation of collagen gels with different stiffness values, stable collagen concentrations, and low cytotoxicity. This method provides a useful system for culturing cells with different stiffness conditions and evaluating the effects of the mechanical environment on cell behavior.

## 3. Conclusions

**Figure 6:**
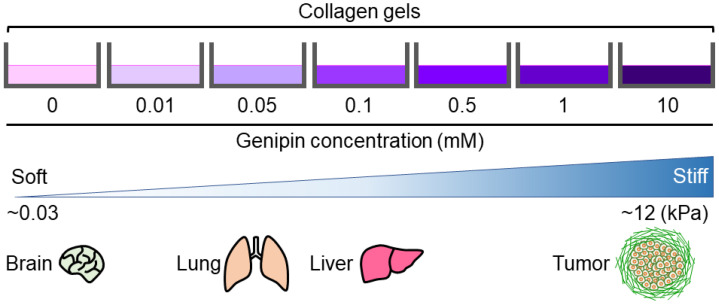
Stiffness-modulation of genipin-mixed collagen gels with the indicated genipin concentrations. The reported stiffness of brain, lung, liver, and tumor is also shown [6,9].

In this study, we established a novel method for preparing cell culture substrates with different stiffness values. Our genipin-mixed collagen gels were substrates of modulated stiffness (0.0292–12.5 kPa) with the same concentration of collagen and low cytotoxicity. This is a good range of stiffness, as we can prepare substrates that mimic soft tissues such as brain tissue, moderate-stiff tissues such as lungs and livers, and abnormally stiff tissues such as tumors in vitro cell culture (Figure 6) [6,9]. Thus, we suggest that our method of preparing collagen gels with different stiffnesses is useful for evaluating the effects of in vivo tissue stiffness on development or cancer progression, contributing to the progress of regenerative medicine and the development of new cancer drugs.

Previous studies have shown the potential of hydrogels as novel therapeutic agents. For instance, hydrogel microspheres are promising therapeutic material for cartilage repair [33], phytomedicine-loaded hydrogels are effective in killing gram-positive and gram-negative bacteria [34], and magnetic hydrogels may be useful for cancer therapy to induce local hyperthermia in tumors [35]. Genipin-mixed collagen gels may also be good therapeutic candidates for preparing biomimetic physical environments for specific tissues and transplanting gels with appropriate cells. Thus, we established a novel method to prepare stiffness-modulated collagen hydrogels for cell culture and potential medical applications.

## 4. Materials and Methods

### 4.1. Gel Preparation

Genipin-mixed collagen gels were prepared under sterile conditions (Figure 1A). First, HEPES buffer (100 mM HEPES, 16 g/L NaCl, 2.3 g/L Na_2_HPO_4_, 0.4 g/L KCl, 0.4 g/L KH_2_PO_4_, pH = 7.3–7.4) and 20 mM genipin (078-03021, Wako, Osaka, Japan) in HEPES buffer were mixed to prepare genipin premix solution (×2 genipin concentration of final collagen gel) on ice. Second, equal volumes of genipin premix solution and Atelocollagen Acidic Solution (5 mg/mL collagen concentration, IPC-50, KOKEN, Tokyo, Japan) were mixed well with gentle pipetting for 60 s on ice. The mixture was then quickly centrifuged to remove bubbles. The solution was poured on an iced dish and incubated at 37 °C in a humidified incubator with 5% CO_2_ for 72 h for gelation. The gels were washed with Dulbecco’s modified Eagle’s medium (DMEM; D6046, Sigma-Aldrich, St. Louis, MO, USA or 08456-65, Nacalai Tesque Inc., Kyoto, japan) for 24 h at 37 °C in a humidified incubator with 5% CO_2_. For MSC culture and growth and survival assay, the gels were washed with phosphate-buffered saline (PBS) for 1 h three times before washing with DMEM. Next, the gels were washed with DMEM supplemented with 10% fetal bovine serum (FBS; 172012, Sigma-Aldrich, St. Louis, MO, USA) and 1% antibiotic/antimycotic solution (A5955, Sigma-Aldrich, St. Louis, MO, USA) for 24 h at 37 °C in a humidified incubator with 5% CO_2_. The gels were then used for cell culture or measurement of the Young’s modulus using atomic force microscopy.

### 4.2. Atomic Force Microscopy (AFM)

Genipin-mixed collagen gels (500 μL) were prepared in a 35 mm plastic dish. AFM indentations for the measurement of surface stiffness were performed using Nanowizard 4 (JPK Instruments, Berlin, Germany) on a TE300 microscope (Nikon Instech, Tokyo, Japan). A 10 μm diameter bead (F8834, Life Technologies, Carlsbad, CA, USA) tip-bound silicon nitride cantilever (MLCT-E, Veeco, Woodbury, NY, USA) with a spring constant of ~0.1 N/m was used. The spring constants of the cantilevers were determined by thermal tuning using a simple harmonic oscillator model. The gels were indented with a calibrated forces of 0.2 nN (gels with 0 mM genipin), 0.5 nN (gels with 0.01, 0,05, or 0.1 mM genipin), 2 nN (gels with 0.5 or 1 mM genipin), or 5 nN (gels with 10 mM genipin) in a scan area of 1 μm^2^ (4 pixels × 4 lines). The Hertz model of impact was used to determine stiffness (Young’s modulus). Three points within the scanning area of each gel were selected, and their corresponding values were used to calculate the average Young’s modulus of each gel. Only the force curves (indentation of at least 100 nm) that fit the Hertz model were used for the calculation. A Poisson’s ratio of 0.5 was used to calculate the Young’s modulus.

### 4.3. Cell Culture

The H1299 human lung cancer cell line (American Type Culture Collection) was cultured in H1299 culture media (DMEM supplemented with 10% FBS and 1% antibiotic/antimycotic solution). Mouse mesenchymal stromal/stem cells (MSCs) [15], kindly gifted by Dr. Suzanne Ponik (University of Wisconsin-Madison), were cultured in MSC culture media (DMEM supplemented with 20% FBS and 1% antibiotic/antimycotic solution). Cells were cultured at 37 °C in a humidified incubator with 5% CO_2_. For collagen coating, plastic or glass dishes were treated with 0.5 mg/mL Atelocollagen Acidic Solution (IPC-50, KOKEN, Tokyo, Japan) or 0.3 mg/mL Cellmatrix Type I-C (Nitta Gelatin, Osaka, Japan; for growth and survival assay). Phase-contrast images were captured using a TE300 microscope (Nikon Instech, Tokyo, Japan) equipped with a 10× objective.

### 4.4. Cell Viability Assay

H1299 cells (1 × 10^5^ cells) or MSCs (2 × 10^5^ cells) were seeded on collagen-coated 35 mm plastic dishes and cultured for 24 h with genipin at the indicated concentrations. The cells were then treated with calcein AM (Dojindo, Mashiki, Japan) and incubated for 1 h. The bright field and fluorescent images of the cells were captured with a ZOE Fluorescent Cell Imager (Bio-Rad Laboratories Inc., Hercules, CA, USA) to count the number of live and total cells. Cell viability (%) was calculated as follows: (number of live cells)/(number of total cells) × 100.

### 4.5. Morphology Assay

H1299 cells (2 × 10^4^ cells) were seeded on 200 μL of genipin-mixed collagen gels in 16 mm glass dishes or collagen-coated 16 mm glass dishes and cultured for 24 h. Then, phase-contrast images of the cells were captured with a TE300 microscope (Nikon Instech, Tokyo, Japan) equipped with a 10× objective. The cell area was calculated using ImageJ software.

### 4.6. Fluorescent Staining

H1299 cells (2 × 10^4^ cells) were seeded on 200 μL of genipin-mixed collagen gels in 16 mm glass dishes or collagen-coated 16 mm glass dishes and cultured for 24 h. Then, the cells were fixed with 4% paraformaldehyde in PBS for 10 min at room temperature and washed three times with PBS. Next, the cells were permeabilized with 0.1% Triton X-100 in PBS for 15 min at room temperature and washed three times with PBS. The cells were blocked with 0.1 or 0.5% bovine serum albumin in PBS for 30–60 min at room temperature. Primary antibody solution (1:250 anti-YAP antibody; 14074S, Cell Signaling Technology Inc., Danvers, MA, USA) in PBS was added and incubated at 4 °C overnight. After three washes with PBS, the secondary antibody solution (1:250 Alexa Fluor 488 goat anti-rabbit; A27034, Invitrogen, Waltham, MA, USA and 1:10,000 Hoechst 33342; H1399, Invitrogen) in PBS was added and incubated at room temperature for 1 h. After three washes with PBS, fluorescent images were captured using an A1 confocal imaging system (Nikon Instech, Tokyo, Japan) with a 60× objective. To quantify the localization of YAP, the fluorescence intensity was calculated as the nuclear/cytosol ratio using ImageJ software.

### 4.7. Differentiation of Mesenchymal Stromal Cells

For smooth muscle differentiation, MSCs (5 × 10^4^ cells) were seeded on 500 μL of genipin-mixed collagen gels in 35 mm plastic dishes or collagen-coated 35 mm plastic dishes and cultured for 24 h with MSC culture media. The medium was then changed to DMEM supplemented with 1% FBS and 1% antibiotic/antimycotic solution for 2 d of culture [36]. For adipogenic conditions, MSCs (8 × 10^4^ cells) were seeded on 250 μL of genipin-mixed collagen gels in 12 well plates or collagen-coated 12 well plates and cultured for 24 h in MSC culture media. Adipogenic differentiation was then performed using the hMSC BulletKit (PT-3004, LONZA, Basel, Switzerland) for 12 d.

### 4.8. qPCR

RNA was extracted using TriPure isolation reagent (Sigma-Aldrich, St. Louis, MO, USA), followed by purification with the FastGene RNA Basic Kit (FG-80250, Nippon Genetics Co., Ltd., Tokyo, Japan). Reverse transcription was performed using the ReverTra Ace qPCR RT Kit (FSQ-201; TOYOBO, Osaka, Japan). qPCR with cDNA was performed using the KAPA SYBR Fast qPCR kit (Kapa Biosystems, Inc., Woburn, MA, USA) and the Applied Biosystems StepOnePlus Real-Time PCR System (Thermo Scientific, Waltham, MA, USA). The following primers (5′-3′) were used: S18 (forward: ACTTTTGGGGCCTTCGTGTC, reverse: GCAAAGGCCCAGAGACTCAT), ACTA2 (forward: CTATTCCTTCGTGACTACTGCCGAG; reverse: GTTATAGGTGGTTTCGTGGATGCCC), ACTG2 (forward: TCGAGTAGCACCAGAAGAGCAC, reverse: CGAATCCAGAACGATGCCTGTG), SMTN (forward: ATGGCAGACGAGGCTTTAGCTG, reverse: TCTCGCTGTTGAGAGTGTAGCC), CEBPA (forward: AACAGCTGAGCCGTGAACTG, reverse: TTTCAGGCCACACCGGAATC), and PPARG (forward: TGATCTTAACTGCCGGATCCAC, reverse: CCCAAACCTGATGGCATTGTG).

### 4.9. Growth and Survival Assay

H1299 cells and MSCs (2 × 10^4^ cells) were seeded on 500 μL of genipin-mixed collagen gels in 35 mm plastic dishes or collagen-coated 35 mm plastic dishes and cultured for 48 h. Nuclei and live cells were stained with Hoechst 33342 (1:4000) and calcein AM (1:1000) in PBS for 30 min, followed by washing with PBS. The nuclei and live cells were imaged using a ZOE Fluorescent Cell Imager (Bio-Rad Laboratories Inc., Hercules, CA, USA). Relative cell growth was evaluated by counting the number of nuclei, with the mean in H1299 cells on 0 mM genipin-mixed collagen gels set as 1). The cell survival (%) was calculated as follows: (number of live cells)/(number of nuclei) × 100.

## Data Availability

All data generated or analyzed during this study are included in this published article and its Supplementary Files.

## Supplementary Materials

The following supporting information can be downloaded from https://www.mdpi.com/article/10.3390/gels9020148/s1 Figure S1. Cell viability of H1299 and mouse mesenchymal stromal cells. Mean ± S.E. *N* = 3 independent experiments. Figure S2. qPCR of SMTN in mouse mesenchymal stromal cells on 0 or 10 mM genipin-mixed collagen gels or collagen-coated plastic substrates with smooth muscle-differentiation condition. S18 was used as an internal control. Mean ± S.D. *N* = 3 independent experiments. * Statistical significance was determined using a 95% confidence interval with Bonferroni correction. Figure S3. (A) Fluorescent staining of nuclei and live H1299 and mouse mesenchymal stromal cells on the 0 or 10 mM genipin-mixed collagen gels or collagen-coated plastic substrates. Scale bar = 100 μm. (B) Relative cell growth and survival of H1299 and mouse mesenchymal stromal cells in (A). Mean±S.D. *N* = 3 independent experiments. N.S.; no statistical significance with Welch’s *t*-test (mesenchymal stromal cells, 10 mM vs plastic) or Student’s *t*-test (the others) with Bonferroni correction.

## References

[1] Discher D.E., Janmey P., Wang Y.L. (2005). Tissue cells feel and respond to the stiffness of their substrate. Science.

[2] Lo C.M., Wang H.B., Dembo M., Wang Y.L. (2000). Cell movement is guided by the rigidity of the substrate. Biophys. J..

[3] Yeung T., Georges P.C., Flanagan L.A., Marg B., Ortiz M., Funaki M., Zahir N., Ming W., Weaver V., Janmey P.A. (2005). Effects of substrate stiffness on cell morphology, cytoskeletal structure, and adhesion. Cell Motil. Cytoskelet..

[4] Haga H., Irahara C., Kobayashi R., Nakagaki T., Kawabata K. (2005). Collective movement of epithelial cells on a collagen gel substrate. Biophys. J..

[5] Meirelles L.D.S., Chagastelles P.C., Nardi N.B. (2006). Mesehcnymal stem cells reside in virtually all post-natal organs and tissues. J. Cell Sci..

[6] Engler A.J., Sen S., Sweeney H.L., Discher D.E. (2006). Matrix elasticity directs stem cell lineage specification. Cell.

[7] Oyama H., Nukuda A., Ishihara S., Haga H. (2021). Soft surfaces promote astrocytic differentiation of mouse embryonic neural stem cells via dephosphorylation of MRLC in the absence of serum. Sci. Rep..

[8] Paszek M.J., Zahir N., Johnson K.R., Lakins J.N., Rozenberg G.I., Gefen A., Reinhart-King C.A., Margulies S.S., Dembo M., Boettiger D. (2005). Tensional homeostasis and the malignant phenotype. Cancer Cell.

[9] Butcher D.T., Alliston T., Weaver V.M. (2009). A tense situation: Forcing tumour progression. Nat. Rev. Cancer.

[10] Ishihara S., Haga H. (2022). Matrix Stiffness Contributes to Cancer Progression by Regulating Transcription Factors. Cancers.

[11] Ishihara S., Yasuda M., Harada I., Mizutani T., Kawabata K., Haga H. (2013). Substrate stiffness regulates temporary NF-κB activation via actomyosin contractions. Exp. Cell Res..

[12] Nukuda A., Sasaki C., Ishihara S., Mizutani T., Nakamura K., Ayabe T., Kawabata K., Haga H. (2015). Stiff substrates increase YAP-signaling-mediated matrix metalloproteinase-7 expression. Oncogenesis.

[13] Wei S.C., Fattet L., Tsai J.H., Guo Y., Pai V.H., Majeski H.E., Chen A.C., Sah R.L., Taylor S.S., Engler A.J. (2015). Matrix stiffness drives epithelial-mesenchymal transition and tumour metastasis through a TWIST1-G3BP2 mechanotransduction pathway. Nat. Cell Biol..

[14] Calvo F., Ege N., Grande-Garcia A., Hooper S., Jenkins R.P., Chaudhry S.I., Harrington K., Williamson P., Moeendarbary E., Charras G. (2013). Mechanotransduction and YAP-dependent matrix remodelling is required for the generation and maintenance of cancer-associated fibroblasts. Nat. Cell Biol..

[15] Ishihara S., Inman D.R., Li W.J., Ponik S.M., Keely P.J. (2017). Mechano-Signal Transduction in Mesenchymal Stem Cells Induces Prosaposin Secretion to Drive the Proliferation of Breast Cancer Cells. Cancer Res..

[16] Shoulders M.D., Raines R.T. (2009). Collagen structure and stability. Annu. Rev. Biochem..

[17] Ishihara S., Mizutani T., Kawabata K., Haga H. (2016). An improved method for western blotting when extracting proteins from mammalian cells cultured on a collagen gel under serum-free conditions. Cytotechnology.

[18] Provenzano P.P., Inman D.R., Eliceiri K.W., Keely P.J. (2009). Matrix density-induced mechanoregulation of breast cell phenotype, signaling and gene expression through a FAK-ERK linkage. Oncogene.

[19] Pelham R.J., Wang Y. (1997). Cell locomotion and focal adhesions are regulated by substrate flexibility. Proc. Natl. Acad. Sci. USA.

[20] Yip A.K., Iwasaki K., Ursekar C., Machiyama H., Saxena M., Chen H., Harada I., Chiam K.H., Sawada Y. (2013). Cellular response to substrate rigidity is governed by either stress or strain. Biophys. J..

[21] Výborný K., Vallová J., Kočí Z., Kekulová K., Jiráková K., Jendelová P., Hodan J., Kubinová Š. (2019). Genipin and EDC crosslinking of extracellular matrix hydrogel derived from human umbilical cord for neural tissue repair. Sci. Rep..

[22] Thomas L.V., Vg R., Nair P.D. (2017). Effect of stiffness of chitosan-hyaluronic acid dialdehyde hydrogels on the viability and growth of encapsulated chondrocytes. Int. J. Biol. Macromol..

[23] Sung H.W., Chang W.H., Ma C.Y., Lee M.H. (2003). Crosslinking of biological tissues using genipin and/or carbodiimide. J. Biomed. Mater. Res. Part A.

[24] Sundararaghavan H.G., Monteiro G.A., Lapin N.A., Chabal Y.J., Miksan J.R., Shreiber D.I. (2008). Genipin-induced changes in collagen gels: Correlation of mechanical properties to fluorescence. J. Biomed. Mater. Res. Part A.

[25] Sung H.W., Huang R.N., Huang L.L., Tsai C.C., Chiu C.T. (1998). Feasibility study of a natural crosslinking reagent for biological tissue fixation. J. Biomed. Mater. Res..

[26] Dupont S., Morsut L., Aragona M., Enzo E., Giulitti S., Cordenonsi M., Zanconato F., Le Digabel J., Forcato M., Bicciato S. (2011). Role of YAP/TAZ in mechanotransduction. Nature.

[27] Jaslove J.M., Nelson C.M. (2018). Smooth muscle: A stiff sculptor of epithelial shapes. Philos. Trans. R. Soc. Lond. B Biol. Sci..

[28] Tamama K., Sen C.K., Wells A. (2008). Differentiation of bone marrow mesenchymal stem cells into the smooth muscle lineage by blocking ERK/MAPK signaling pathway. Stem Cells Dev..

[29] Gu W., Hong X., Le Bras A., Nowak W.N., Issa Bhaloo S., Deng J., Xie Y., Hu Y., Ruan X.Z., Xu Q. (2018). Smooth muscle cells differentiated from mesenchymal stem cells are regulated by microRNAs and suitable for vascular tissue grafts. J. Biol. Chem..

[30] Nagayama M., Uchida T., Gohara K. (2007). Temporal and spatial variations of lipid droplets during adipocyte division and differentiation. J. Lipid Res..

[31] Rosen E.D., Hsu C.H., Wang X., Sakai S., Freeman M.W., Gonzalez F.J., Spiegelman B.M. (2002). C/EBPalpha induces adipogenesis through PPARgamma: A unified pathway. Genes Dev..

[32] Lai J.Y., Ma D.H., Lai M.H., Li Y.T., Chang R.J., Chen L.M. (2013). Characterization of cross-linked porous gelatin carriers and their interaction with corneal endothelium: Biopolymer concentration effect. PLoS ONE.

[33] Lin F., Li Y., Cui W. (2023). Injectable hydrogel microspheres in cartilage repair. Biomed. Technol..

[34] Das P., Ganguly S., Saravanan A., Margel S., Gedanken A., Srinivasan S., Rajabzadeh A.R. (2022). Naturally Derived Carbon Dots In Situ Confined Self-Healing and Breathable Hydrogel Monolith for Anomalous Diffusion-Driven Phytomedicine Release. ACS. Appl. Bio Mater..

[35] Ganguly S., Margel S. (2021). Design of Magnetic Hydrogels for Hyperthermia and Drug Delivery. Polymers.

[36] Park J.S., Chu J.S., Tsou A.D., Diop R., Tang Z., Wang A., Li S. (2011). The effect of matrix stiffness on the differentiation of mesenchymal stem cells in response to TGF-β. Biomaterials.

