# Supplementary material for: Stiffness-Modulation of Collagen Gels by Genipin-Crosslinking for Cell Culture
Source: Gels. 2023 Feb 10;9(2):148. doi: 10.3390/gels9020148 (PMC9957210; doi:10.3390/gels9020148)
Supplement: Supplementary file 1 [file gels-09-00148-s001.zip › gels-2158340-supplementary.pdf]

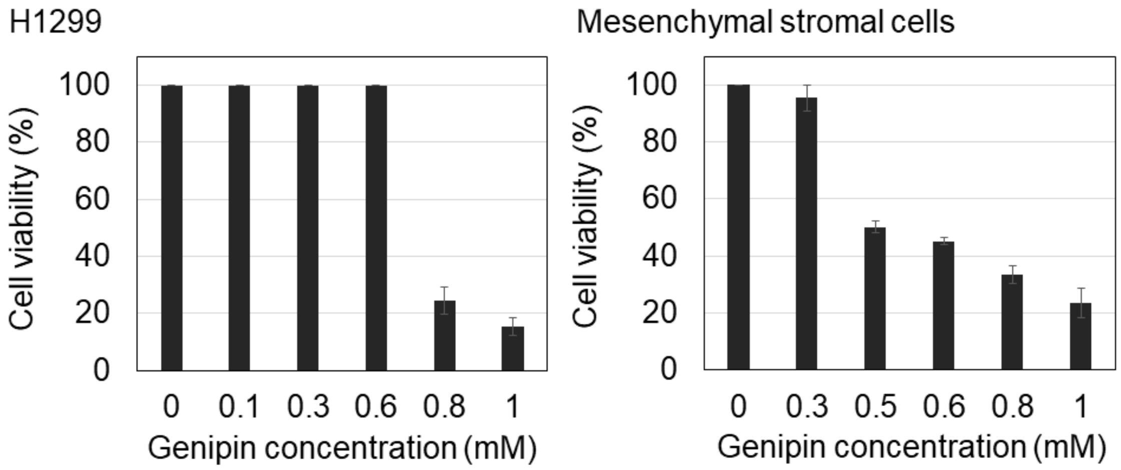

Figure S1. Cell viability of H1299 and mouse mesenchymal stromal cells. Mean±S.E. $N = 3$ independent experiments.

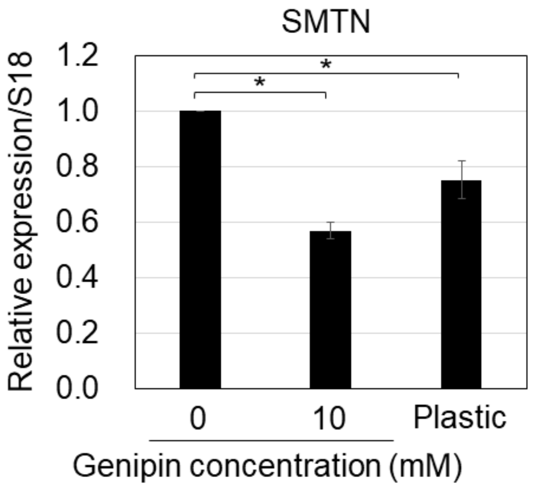

Figure S2. qPCR of SMTN in mouse mesenchymal stromal cells on 0 or 10 mM genipin-mixed collagen gels or collagen-coated plastic substrates with smooth muscle-differentiation condition. S18 was used as an internal control. Mean±S.D. $N = 3$ independent experiments. *Statistical significance was determined using a 95% confidence interval with Bonferroni correction.

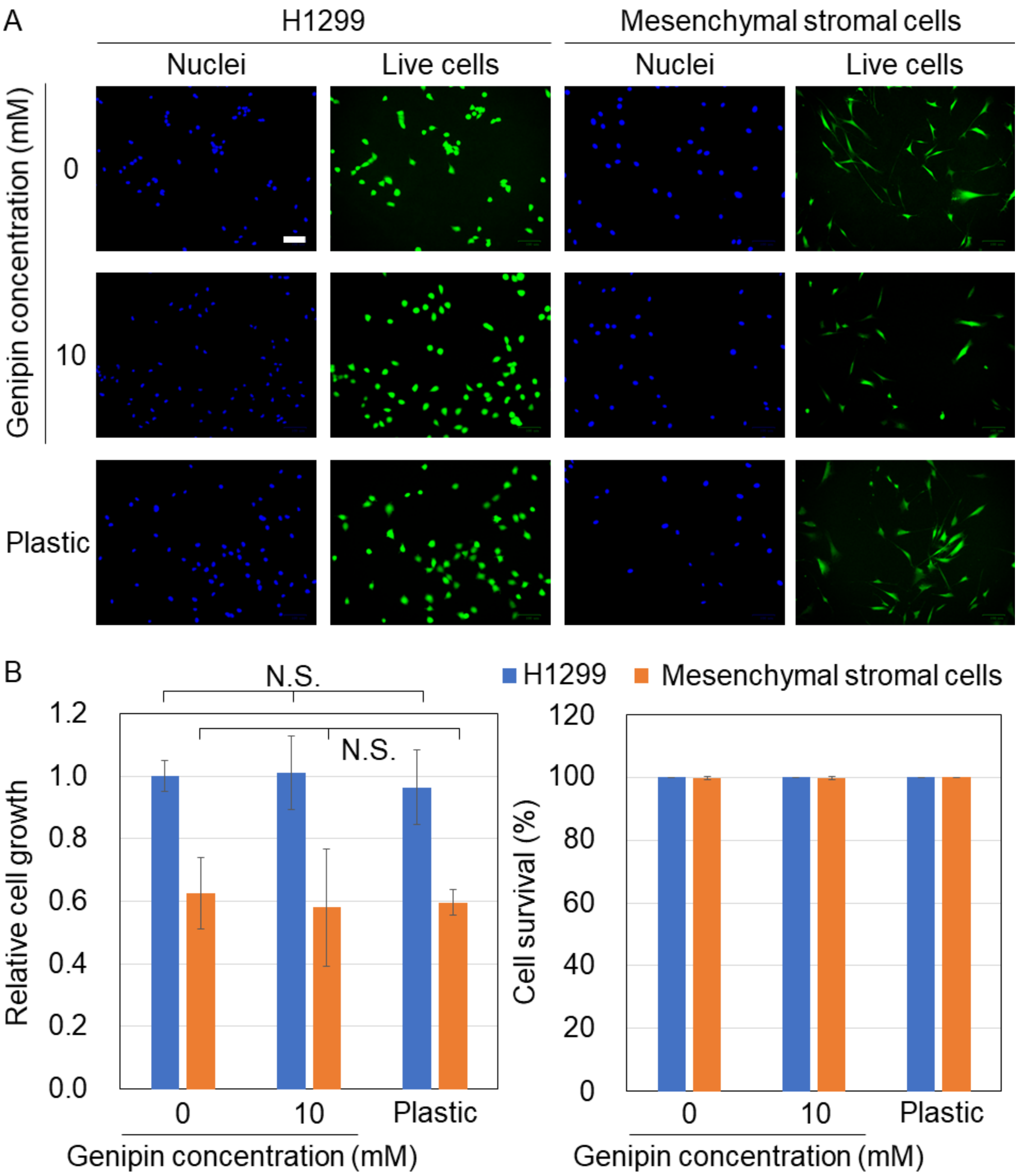

Figure S3. (A) Fluorescent staining of nuclei and live H1299 and mouse mesenchymal stromal cells on the 0 or 10 mM genipin-mixed collagen gels or collagen-coated plastic substrates. Scale bar = 100 µm. (B) Relative cell growth and survival of H1299 and mouse mesenchymal stromal cells in (A). Mean±S.D. N = 3 independent experiments. N.S.; no statistical significance with Welch's t-test (mesenchymal stromal cells, 10 mM vs plastic) or Student's t-test (the others) with Bonferroni correction.
